# Importance of Longitudinal Assessments in a Case of Comorbid Polysubstance Use Disorder and Borderline Personality Disorder Misdiagnosed As Bipolar I Disorder

**DOI:** 10.7759/cureus.45253

**Published:** 2023-09-14

**Authors:** Esther Tsyngauz, Andrew K Chiu, Zeeshan Faruqui

**Affiliations:** 1 Psychiatry and Behavioral Sciences, Drexel University College of Medicine, Philadelphia, USA; 2 Psychiatry, Drexel University College of Medicine, Philadelphia, USA; 3 Interventional Psychiatry, Keystone Health, Chambersburg, USA

**Keywords:** self-harm, depression, substance use disorder (sud), borderline personality disorder, bipolar affective disorder

## Abstract

Differentiating between borderline personality disorder (BPD) and bipolar disorder (BD) can be difficult. Both may present with altered mood states, deliberate self-harm, suicidality, impulsivity, unstable relationships, and risky behaviors. A manic episode is characterized by at least one week of elevated or irritated mood and at least three of the following: distractibility, impulsivity, grandiosity, flight of ideas, psychomotor activity, decreased need for sleep, and pressured speech. Borderline personality disorder is characterized by unstable mood and relationships, fear of abandonment, impulsivity, self-mutilation, suicidality, and a feeling of emptiness. In combination with polysubstance use, borderline personality disorder can present similarly to a manic episode and lead to an incorrect diagnosis of bipolar I disorder. In this study, we present a 44-year-old female whose psychiatric history highlights the importance of long-term patient observation in making an accurate diagnosis. Over the course of several years, she was given incorrect psychiatric diagnoses, including attention deficit hyperactivity disorder (ADHD), generalized anxiety disorder, and bipolar I disorder. As a result, her interpersonal relationships remained unstable and significantly affected her quality of life. Over the course of consistent, long-term psychiatric appointments, conversations with family members, and notes from previous psychiatrists, it became evident that substance use had also complicated her psychiatric history, leading to the aforementioned diagnoses. Once this was established, she was diagnosed with borderline personality disorder; subsequent correct medical intervention has been integral in helping her maintain a steady job and improve her interpersonal relationships and quality of life.

## Introduction

Borderline personality disorder and bipolar disorder share multiple similarities. Distinguishing between the two is critical in making a proper diagnosis. Both can present with altered mood states, deliberate self-harm, suicidality, impulsivity, unstable relationships, and risky behaviors. According to the Diagnostic and Statistical Manual of Mental Disorders Fifth Edition, Text Revision (DSM-5-TR), a manic episode is characterized by ≥one week of elevated or irritated mood and ≥three of the following: distractibility, impulsivity, grandiosity, flight of ideas, psychomotor activity, decreased need for sleep, and pressured speech [1] and borderline personality disorder is characterized by unstable mood and relationships, fear of abandonment, impulsivity, self-mutilation, suicidality, and a feeling of emptiness [2].

The diagnostic difficulty between borderline personality disorder and bipolar disorder is well-known [3]. With no biological markers to distinguish between the two, it is critical to elucidate an accurate history of the patient directly and corroborate with family, friends, previous physicians, and the patient’s medical record. There has also been an alternative DSM-5 model considered for personality disorders. It includes a dimensional rating of the severity of impairment in personality functioning and 25 personality traits that have been organized into five domains [4]. This model gives an alternative method to assess personality disorders and allows physicians to use a step-wise approach to diagnose personality disorders. The prevalence of borderline personality disorder in the general population is 1.6%, compared to 20% of the inpatient population [5]. The prevalence of bipolar disorder is 1% in the general population [5].

Borderline personality disorder is a personality disorder that consists of “pervasive, enduring patterns of thinking, perceiving, reacting, and relating that cause significant distress or functional impairment” [6]. The etiology of borderline personality disorder is multi-factorial. Many patients have a history of childhood trauma, including physical and sexual abuse, neglect, separation from caregivers, or loss of a parent [7]. There is also a genetic component, as first-degree relatives of patients with borderline personality disorder have a five times increased risk of developing the disorder [7]. According to the DSM-5, the symptoms have to begin by early adulthood and must include five or more of the following: (1) frantic efforts to avoid real or imagined abandonment, (2) a pattern of unstable and intense interpersonal relationships characterized by alternating between extremes of idealization and devaluation, (3) identity disturbance - markedly and persistently unstable self-image or sense of self, (4) impulsivity in at least two areas that are potentially self-damaging (spending, substance abuse, reckless driving, unsafe sex, binge eating, etc.), (5) affective instability caused by marked reactivity of mood, (6) chronic feelings of emptiness, (7) inappropriate, intense anger, or difficulty controlling anger, (8) transient paranoid ideation or severe dissociative symptoms [7].

Borderline personality disorder begins in early adulthood and affects every aspect of a patient’s life. It can be very destructive and lead to significant problems in a patient’s education, interpersonal relationships, and career. A borderline personality disorder is one of the most common psychiatric problems, and studies have shown that up to 50% of patients with repetitive suicide attempts in emergency rooms have the disorder [8]. Even with this high prevalence, diagnosing borderline personality disorder remains difficult. This could be because many of the symptoms associated with borderline personality disorder (BPD) are problems in interpersonal functioning, which can be difficult to deduce during a psychiatric appointment [8]. Additionally, there are not many pharmacological tools to treat patients with BPD, and many physicians are hesitant to diagnose a patient with a disorder that has a negative stigma [8]. These reasons lead to patients being misdiagnosed and not receiving the proper treatment.

Use of substances can further complicate the diagnosis of BPD. According to a review of studies that compare the prevalence of substance use disorders in patients with BPD, the prevalence has been as high as 53.19% [9]. For cocaine use, the prevalence was between 13.8 and 39%, and for opioid dependence, the prevalence was between 11.5 and 51% [9]. The potentially significant percentage of co-occurrence between substance use and BPD can make diagnosing the disorder even more difficult. For example, when substances such as cocaine can cause impulsivity in otherwise healthy individuals, concurrent substance use can easily complicate establishing the diagnosis of borderline personality disorder.

The McLean Screening Instrument is a valuable tool for identifying and diagnosing borderline personality disorder [10]. A score of seven or higher indicates a high chance that a patient will meet the criteria for BPD [10].

Bipolar disorder (BD) and borderline personality disorder (BPD) have many overlapping features, including mood alteration, self-harm, suicidality, and impulsivity [11]. Making an accurate diagnosis is paramount, particularly with BD, as untreated BD is associated with mortality by suicide up to 20 times that of the general population [12].

This case highlights the importance of consistent psychiatric care, which can help parse the nuances of both conditions and lead to more accurate diagnoses. The patient went through years of misdiagnoses, constant transitions to new psychiatrists, and multiple unsuccessful trials on numerous medications. Once she was correctly diagnosed, she was able to have a more stable lifestyle and meaningful relationships. With longitudinal care and more intentional history-taking, patients can be correctly diagnosed earlier and have a significantly improved quality of life.

## Case presentation

We present a case of a 44-year-old female with a complicated psychiatric history. She has had multiple inpatient psychiatric hospitalizations, an extensive drug use history, and frequent changes in psychiatrists, which made determining a correct diagnosis more difficult. She had been previously diagnosed with bipolar disorder, attention deficit hyperactivity disorder, and generalized anxiety disorder, all before being correctly diagnosed with borderline personality disorder. She first presented to the current psychiatrist in 2019 after being hospitalized for suicidal ideation. She had a basic metabolic panel (BMP), complete blood count (CBC), urine drug screen, lithium level, and vitamin D level checked. Her BMP, CBC, lithium level, and vitamin D level were normal. The urine drug screen was positive for tetrahydrocannabinol (THC) and benzodiazepines. Her physical examination was unremarkable, except for a BMI of 32.

**Figure 1:**
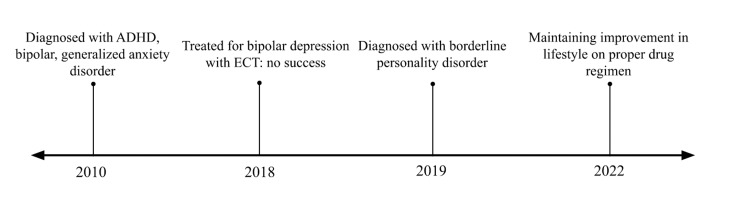
Timeline of patient's psychiatric diagnoses. ADHD: attention-deficit/hyperactivity disorder; ECT: electroconvulsive disorder The image is created by the authors (ET and AC) of this study.

The patient was raised by a single mother, who had borderline personality disorder and significant alcohol use disorder. The patient was neglected and abused by the mother’s different male partners. As she grew up, she had impulsive sexual relationships with men. Her traumatic childhood significantly affected her behavior and this trauma is inevitably intertwined with borderline personality disorder. In her 20s, the patient's substance use became severe. She began using lysergic acid diethylamide (LSD), 3,4-methylenedioxy​methamphetamine (MDMA), marijuana, and psilocybin. She continued to use drugs while denying substance use during her psychiatric appointments. She had multiple psychiatric hospitalizations for suicidal ideation, episodes of paranoia, and attempted overdose. She also had a history of multiple misdemeanors, including numerous instances of driving under the influence. It took many years to diagnose the patient with borderline personality disorder and determine the proper drug regimen (Figure 1). In 2010, she was diagnosed with attention deficit hyperactivity disorder (ADHD), bipolar, and generalized anxiety disorder with polysubstance abuse. In 2018, she was treated for bipolar depression with electroconvulsive therapy. She was treated three times a week for four weeks with no improvement. Over the years, she had failed multiple medication trials including mood stabilizers, such as oxcarbazepine; antidepressants, such as milnacipran and vilazodone; antipsychotics, such as risperidone, ziprasidone, and aripiprazole; and other medications, including propranolol. None were effective in addressing her symptoms, though they led to an array of adverse effects. Mood stabilizers were prescribed to decrease her symptoms of impulsivity, antidepressants were tried to improve her mood, and antipsychotics were tried for impulsivity and anxiety. All these medications were titrated to the optimal dose that the patient could tolerate. Oxcarbazepine, which works by binding to sodium channels, has been shown to improve symptoms of anxiety and anger and to help with interpersonal relationships. Similarly, propranolol, a beta-blocker, was trialed to ameliorate her anxiety and aggression. The patient reported that neither milnacipran, which inhibits reuptake of serotonin and norepinephrine, nor vilazodone, which works by increasing serotonin, improved her depressive symptoms. However, they caused increased irritability and gastrointestinal upset, respectively. Risperidone, which inhibits reuptake of serotonin and norepinephrine, did not cause an improvement in impulsivity or depressive symptoms. It caused weight gain, muscle spasms, and increased sedation. Ziprasidone led to excessive sedation and aripiprazole led to weight gain and irritability. Oxcarbazepine resulted in no improvements in depression and anxiety, and propranolol caused dizziness. She continued to have unstable interpersonal relationships, employment status, and housing situations. She became a patient of the office when she presented with self-reported symptoms of impulsivity, poor frustration tolerance, unstable relationships, and self-mutilative acts. Until 2019, she had not been diagnosed with borderline personality disorder and her symptoms aligned with both borderline personality disorder and bipolar I disorder.

In 2019, she was finally diagnosed with borderline personality disorder by her current psychiatrist. As mentioned earlier, she was a heavy polysubstance user. She used both stimulants and synthetic marijuana. She impulsively decided to move several states away with her partner to start a business; friends, family, and doctors lost contact with her for several months. Eventually, she reached out for help after the couple spent all their money on recreational substances. She revealed that she was being trafficked and was living in Alabama. Her initial plan was to travel to Colorado with her boyfriend and start a marijuana business. This instance, combined with the patient’s self-reported symptoms, would appear to satisfy the DSM-5 criteria for a manic episode. She impulsively left her hometown to move across the country and had a distinct period of impulsivity evidenced by her attempt to open a business with no experience. This period lasted over a week and was characterized by elevated mood, grandiosity, decreased need for sleep, and impaired goal-directed activity. With this information, it would have been reasonable to arrive at a diagnosis of bipolar I disorder. However, this spontaneous decision, combined with her self-reported symptoms of labile relationships, emotional reactivity, unstable self-image, extreme moodiness, distrust of others, and self-harm also add up to seven points on the McLean Screening Instrument, which has a sensitivity of 81% and specificity of 89% (Table 1) [13]. The cut-off score on the McLean Screening Instrument is seven, with a maximum score of 10. This is highly suggestive of borderline personality disorder [10]. This depicts the complexity of determining the correct diagnosis for this patient.

**Table 1:** McLean Screening Instrument is a useful tool to assist in diagnosing borderline personality disorder.

McLean Screening Instrument	Point
Have any of your closest relationships been troubled by a lot of arguments or repeated breakups?	1
Have you deliberately hurt yourself physically?	1
Have you had at least two other problems with impulsivity (eating binges, spending sprees, drinking too much, and verbal outbursts)?	1
Have you been extremely moody?	1
Have you felt very angry a lot of the time? How about often acting in an angry or sarcastic manner?	1
Have you often been distrustful of other people?	1
Have you frequently felt unreal or as if things around you were unreal?	1
Have you chronically felt empty?	1
Have you often felt that you have no idea of who you are or that you have no identity?	1
Have you made desperate efforts to avoid feeling abandoned or being abandoned (repeatedly called someone to reassure yourself that he or she still cared, begged them not to leave you, clung to them physically)?	1

**Figure 2:**
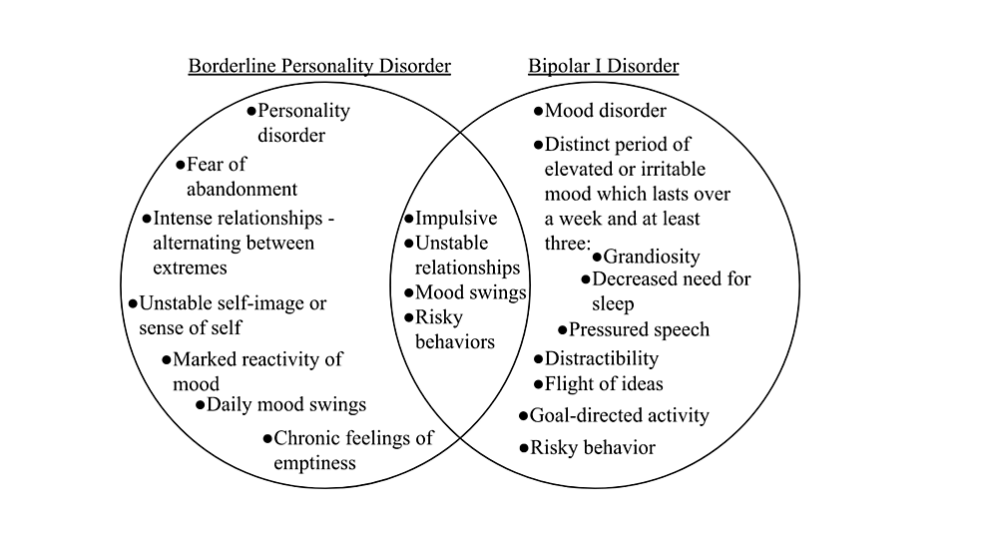
Comparison of the differences and similarities between borderline personality disorder and bipolar I disorder. The image is created by the authors (ET and AC) of this study.

Subsequent psychiatric visits revealed her “episodes” were planned months in advance. Substance use was involved in the creation and execution of the plan to go to Alabama. The patient’s actions were heavily influenced by substance abuse, which contributed to her symptoms of grandiosity, goal-directed activity, and risky behavior that were initially presumed to be part of a manic episode. Without that added information, it would have been difficult to differentiate between bipolar I and borderline personality disorders, as they have many similarities (Figure 2). It would have been reasonable to categorize her most recent “episode” as mania; however, given the context of the patient’s decisions in the setting of extensive substance use, BPD is likely the more appropriate diagnosis. Further review of her chart using previous psychiatrists’ notes and speaking to family members illustrated that her mood reactivity fluctuates throughout the day and does not stay elevated for days at a time, adding support for the BPD diagnosis. The most successful drug regimen to date for this patient is venlafaxine, buprenorphine, and bupropion. She has also successfully taken part in dialectical behavioral therapy (DBT) through individual therapy. Her DBT sessions were two to three times weekly. Her lifestyle has seen dramatic changes since being correctly diagnosed with borderline personality disorder. Her relationships have improved and are more stable. She has been able to maintain a job and her diagnosis helps her understand her actions and validates her past experiences. She follows up with the current psychiatrist once a month. She is currently taking venlafaxine 150 mg daily and has DBT sessions weekly.

## Discussion

This study illustrates the importance of using long-term care to distinguish between borderline personality disorder and bipolar I disorder. Patients with BPD have poor frustration tolerance which can lead to the physician feeling overwhelmed and the patient feeling dissatisfied. This can cause disjointed care as the patient switches from psychiatrist to psychiatrist. It is important to observe the patient over a significant amount of time to help elucidate the correct diagnosis. In addition to observation, it is essential to be intentional in gathering collateral information from people in the patient’s life: family members, friends, and previous psychiatrists. With the newer electronic medical record (EMR) system, we were able to use Care Everywhere to find notes from previous psychiatrists and gain a greater understanding of the patient’s long-term psychiatric history. Third-party information is critical and provides more context to the patient’s condition than singular emergency department visits or brief psychiatric hospitalization. Identifying the correct diagnosis allows for proper pharmacotherapy and gives patients with borderline personality disorder the opportunity to better understand their patterns, thought processes, and relationships.

Borderline personality disorder and bipolar disorder have been known to cause diagnostic confusion. Although they can both present with altered mood states, deliberate self-harm, suicidality, impulsivity, unstable relationships, and risky behaviors, they are different disorders. Borderline personality has “transient mood shifts” that occur due to stressors, while patients with bipolar disorder have sustained mood periods that last for multiple days at a time [14]. Studies have also shown that patients with BPD switch from euthymia to anger, while those with bipolar disorder switch from depression to elation [14]. Other ways to differentiate between BPD and BD include a family history of bipolar emotional dysregulation compared to mood episodes, pattern of unstable relationships, absence of hypomania or mania, and response to medications [14]. These are crucial factors to differentiate between two seemingly very similar disorders. According to a systematic review comparing borderline personality disorder and bipolar I disorder, patients with BPD have more negative attitudes toward others, more unstable interpersonal relationships, and more impulsiveness [14]. Both disorders overlap in symptoms of depression, dysphoria, suicidal ideation, and childhood trauma [14]. To gather enough information to make an accurate diagnosis, it is important to talk to the patient over time, as well as their friends, family, and previous doctors.

Correctly diagnosing someone with borderline personality disorder has a significant impact on the patient's life. Patients with BPD have higher rates of mortality and shorter lifespans compared to the rest of the population [14]. The suicide rate is as high as 10% [15]. The correct diagnosis and treatment can have profound effects on patients' lives, but there is a debate on whether to disclose the diagnosis of BPD to patients. There are multiple reasons for this. One of them is that mental health professionals believe that patients with BPD overutilize the healthcare system [15]. The negative stigma associated with BPD by both health professionals and the general population leads to patients not being informed about their diagnosis [14]. There is also debate on the effectiveness of treatment and some physicians feel there would be no benefit in disclosing the diagnosis [15]. The use of pharmacotherapy should be individualized to the patient. The research on pharmacotherapy for borderline personality disorder has not shown a strong indication for pharmacotherapy [16]. The research studies have been limited by small sample size and short trials [16]. Meta-analyses have shown that mood stabilizers can reduce anger and impulsivity, while antidepressants may play a role in improving mood [16]. However, informing patients of their diagnosis can significantly change their quality of life. A formal diagnosis helps patients validate their experiences and connect with others who have had similar experiences [15].

There are various psychotherapies that are available for patients with borderline personality disorder. These include cognitive behavioral therapy, transference-focused psychotherapy, and systems training for problem-solving [16]. The most widely used is dialectical behavioral therapy. Dialectical behavior therapy (DBT) includes individual therapy, group therapy, group, skills training, telephone coaching, and a telephone consultation team [17]. This therapy has been shown to be effective in decreasing suicide risk and suicide attempts in patients with BPD [17]. Another study that analyzed three randomized control trials of women who participated in DBT for a year and had one year of follow-up, showed a decrease in suicide attempts, depression, and control of anger over time [18]. It has also been shown to reduce suicidal ideation, severity of self-harm, and improve reasons for living [17]. The efficacy of DBT further highlights the importance that patients with BPD benefit from proper treatment, which is only possible with a correct diagnosis.

## Conclusions

Diagnosing a patient correctly can have a profound impact on a patient’s quality of life and disease course. With concurrent substance use, it becomes increasingly difficult to correctly diagnose a patient and provide them with correct therapy. This study illustrates the importance of obtaining information from various sources to better understand the patient and how consistent, longitudinal care is key to making an accurate diagnosis. Additionally, when a patient is more accepting of their diagnosis, they are more able to participate in their treatment and thus have increased treatment adherence and better outcomes. Physicians need to take time to evaluate the patient and encourage active participation in their treatment. They should also use appropriate scales to determine an accurate diagnosis. Dialectical behavior therapy helps patients to better understand their emotions, thoughts, and actions. Many patients use psychotherapy, which gives them a space to express themselves without judgment and can provide some relief. The combination of pharmacotherapy and psychotherapy can help patients more than pharmacotherapy alone.
